# Physical Frailty and Amyloid-β Deposits in the Brains of Older Adults with Cognitive Frailty

**DOI:** 10.3390/jcm7070169

**Published:** 2018-07-09

**Authors:** Dong Hyun Yoon, Jun-Young Lee, Seong A Shin, Yu Kyeong Kim, Wook Song

**Affiliations:** 1 Health and Exercise Science Laboratory, Institute of Sports Science, Seoul National University, 599 Gwanangno, Gwanak-Gu, Seoul 08826, Korea; ycool14@snu.ac.kr; 2 Department of Psychiatry and Behavioral Science, SMG-SNU Boramae Medical Center 5-20 Boramaero, Dongjak-Gu, Seoul 07061, Korea; benji@emocog.com; 3 Department of Psychiatry and Behavioral Science, Seoul National University College of Medicine, 101 Daehak-ro, Jongno-gu, Seoul 03080, Korea; 4 Department of Nuclear Medicine, Seoul National University College of Medicine, SMG-SNU Boramae Medical Center, 5-20 Boramaero, Dongjak-Gu, Seoul 07061, Korea; sshi082@snu.ac.kr; 5 Department of Biomedical Science, Seoul National University College of Medicine, Seoul National University College of Medicine, 101 Daehak-ro, Jongno-gu, Seoul 03080, Korea; 6 Department of Nuclear Medicine, Seoul National University College of Medicine, 101 Daehak-ro, Jongno-gu, Seoul 03080, Korea; 7 Institute on Aging, Seoul National University, Seoul 03087, Korea

**Keywords:** cognition, frailty, physical function, brain aging

## Abstract

Background: Cognitive frailty and impairment are phenotypically and pathophysiologically correlated with physical frailty. We examined associations between accumulation of amyloid-β in the brain as a brain imaging biomarker and phenotypes of physical frailty (weight loss, weakness, exhaustion, slowness, low physical activity) in older adults with mild cognitive impairment (MCI) and cognitive frailty. Methods: Cross-sectional associations between brain amyloid-β accumulation measured with ^11^C-Pittsburgh compound B (PiB)-positron emission tomography (PET) and physical frailty were examined in 48 elderly participants (mean age: 75.1 ± 6.6 years; 73% female). Cortical and regional standard uptake value ratios (SUVRs) were obtained. Main outcome measures included frailty phenotypes and physical functions (gait speed, short physical performance battery, and Timed Up and Go tests). Results: Mean cortical region of interest and regional SUVRs (frontal cortex, temporal cortex, parietal cortex, precuneus/posterior cingulate cortex (PC/PCC), hippocampus, basal ganglia, and global SUVR) were associated with gait speed, Timed Up and Go, and short physical performance battery (PC/PCC, basal ganglia). In addition, SUVRs of all brain regions were significantly linked to weakness. Conclusion: SUVRs of all brain regions revealed an association between brain amyloid-β accumulation and weakness. Furthermore, global SUVRs (frontal cortex, temporal cortex, parietal cortex, PC/PCC, hippocampus, basal ganglia) were associated with gait parameters.

## 1. Introduction

Frailty is a retrievable pathological aging process. It occurs at an intermediate stage between age-related diseases and poor prognosis [1]. Typically, physical frailty is defined by the presence of at least three of the following five criteria: fatigue, poor muscle strength, slow gait, diminished physical activities, and unintentional weight loss. Pre-physical frailty is determined by the presence of one or two of these five criteria [2]. Phenotypes of frailty and cognitive impairment are correlated with each other, sharing several pathophysiological mechanisms with physical frailty. The concept of “cognitive frailty” was proposed to stimulate additional research in this area, emphasizing the key role of brain aging [3,4]. Cognitive frailty is a heterogeneous clinical syndrome occurring in elderly individuals, excluding those with Alzheimer’s disease (AD) and other dementias. It is characterized by concurrent physical frailty and potentially reversible cognitive impairment (clinical dementia rating score (CDR) = 0.5) [3]. Therefore, cognitive frailty is a type of pathological brain aging and a precursor of neurodegenerative processes.

Although physical frailty and cognition are known to be associated with each other, causal links between physical frailty and cognitive impairment are currently unclear [1]. Furthermore, information about the correlation between physical frailty and biomarkers of AD in humans is limited, particularly regarding amyloid-β (Aβ) accumulation in the brain. Previous studies have suggested that individuals who are physically active [5,6,7] with increased gait speed [8,9] have significantly lower Aβ deposition compared to inactive people with poor gait [10]. Moreover, the presence of β-amyloid peptides in muscle fibers is associated with inclusion body myositis and reduced muscle strength [11]. However, to the best of our knowledge, no study has reported the association between physical frailty and amyloid-β accumulation in the brain using 11C-Pittsburgh compound B (PiB)-positron emission tomography (PET) to determine their roles in frailty status under a dose-response relationship. Therefore, the objective of this study was to examine the association between amyloid-β accumulation in the brain using a brain imaging biomarker and physical frailty (weight loss, weakness, exhaustion, slowness, low physical activity) in older adults with mild cognitive impairment (MCI) and cognitive frailty. We hypothesized that amyloid-β accumulation in the brain was negatively associated with physical frailty status.

## 2. Materials and Methods

### 2.1. Study Sample

Fifty-nine subjects participating in the Korean Brain Aging Study for Early Diagnosis and Prediction of Alzheimer’s Disease (KBASE) were selected for this study. Inclusion criteria were as follows: (1) individuals who were 65 years of age and older; (2) lived in Seoul; (3) had no history of depression, degenerative neurologic disease, hospital admission in the past 12 months for any reason, illiteracy, or dementia; (4) could walk at least 10 m independently without using a mobility aid; and (5) had a CDR of 0.5. Of these 59 subjects who underwent PiB-PET with cognitive and physical performance tests included in our analyses, 11 were excluded because they had low cognitive function (i.e., CDR >1.0) or refused CDR measurement. The remaining 48 subjects, with a mean age of 75.1 ± 6.6 years (73% female), were selected to participate in this study (Table 1). Physical frailty status was determined according to the 5 criteria defining physical frailty suggested by the Cardiovascular Health Study (CHS) [3]: unintentional weight loss, slowness, weakness, exhaustion, and low activity. Each criterion was scored 1 if it was present or 0 if absent. A total cumulative score ranging from 0 to 5 was used to classify participants as robust (score = 0), pre-frail (1–2), or frail (3–5). Cognitive frailty was defined as the simultaneous presence of physical frailty as described above and cognitive impairment with a CDR of 0.5 and the absence of concurrent dementia [3]. The study protocol was approved by the Institutional Review Board (IRB) of the Seoul Metropolitan Government and Seoul National University (SNG-SNU) Boramae Center, South Korea. All participants provided written informed consent (approval number: SNUH IRB No. 26-2015-60).

### 2.2. Measurements

#### 2.2.1. Frailty Definition

Frailty was assessed according to the previous criteria [12] with 5 components. Shrinking/weight loss was operationally defined if the participant self-reported an unintentional weight loss of 4.5 kg in the last 12 months or if the body mass index was less than 18.5 km/m^2^. Weakness was defined by low grip strength, which was adjusted by gender and body mass index. Grip strength was measured using a hand-to-hand dynamometer (Takei Scientific Instruments, Niigata, Japan). This assessment protocol was repeated 4 times with a break in between. The average grip strength was recorded in kilograms and a cutoff point was adopted from the criteria of the Cardiovascular Health Study. Exhaustion was defined based on answers to 2 questions on the Center for Epidemiologic Studies–Depression (CES-D) scale: “How often have you ever felt that everything you had done was useless in the last week?” and “How often have you ever felt that everything you had to do was not done in an appropriate mood last week?” Exhaustion was indicated if the response was “most of the time” or “often” [13]. Slowness was defined as a low gait speed over a distance of 4 m. Detailed measurement of gait speed was described in Jung et al. (2016), and a speed of <0.8 m/s indicated frailty-related slowness [14]. Low physical activity corresponded to responses to items on the International Physical Activity Questionnaire (IPAQ) concerning low, middle, and high levels of physical activity. Responses describing low physical activity were indicative of frailty [4]. Participants were classified as “frail,” “pre-frail,” or “robust” when ≥3 criteria, 1 or 2 criteria, or no criteria were met, respectively.

#### 2.2.2. Functional Performance

A short physical performance battery (SPPB) was used to assess balance (ability to stand with the feet together in side-by-side, semi-tandem, and tandem positions), gait speed (usual time to walk 4 m), and 5 chair-stand tests (time to rise from a chair and return to a seated position 5 times without using arms). Each test received a performance score, with a possible total of 12 points comprising balance test (4 points), 4 m gait speed test (4 points), and chair-stand test (4 points). In the chair-stand test, the participant was initially seated, and upon verbal command, the participant stood and sat 5 times. A stopwatch was used to record the time (in seconds) to complete the task. Balance was measured in 3 tests following an explanation. In the side-by-side stand test, feet were positioned together and balance was maintained for 10 s. In the semi-tandem stand test, the participant stood with a toe of the dominant foot touching the middle of the opposite foot for 10 s. In the tandem stand test, the participant stood with a toe of the dominant foot touching the heel of the opposite foot for 10 s. The 4 m gait test was used to assess gait speed. The average time of 2 trials in the walking speed test was recorded. Three individual categorical scores were added to obtain a summary performance score for each participant (score range, 0–12), with higher scores indicating better lower body function. A change in SPPB score of 1 point was considered significant [15,16].

The Timed Up and Go (TUG) test is a widely used method to evaluate basic mobility maneuvers. The TUG protocol used in this study was based on a previous study [16]. It was performed as follows. Upon hearing a signal from a single rater, the subject rose from the chair without using armrests, walked 2.44 m in a linear path as quickly as possible, turned around at a marker, walked back to the chair, and sat down.

#### 2.2.3. Neuropsychological Battery

A sensitive and validated neuropsychological test battery was used to assess each participant’s cognitive function. The neuropsychological battery included the Korean version of the Consortium to Establish a Registry for Alzheimer’s Disease (CERAD-K), the Korean version of the Mini–Mental State Examination (MMSE-K) [17], and CDR scales. A single rater evaluated these for all participants. The MMSE-K is commonly utilized to screen for dementia. It consists of 11 questions and tasks in 5 cognitive domains: orientation (10 points), memory (6 points), attention (5 points), language ability (7 points), and comprehension/judgment (2 points). The highest possible score is 30, with higher scores indicating higher levels of cognitive function. The CERAD-K comprises 5 subtests derived from previously established cognitive tests: an executive domain of verbal fluency test (0–24 points), a language domain of Boston Naming Test (BNT) (0–15 points), a memory domain of Word-List-Learning test (0–30 points) with delayed recall (0–10 points) and recognition (0–10 points), and a visuospatial domain of visual construction test (0–11 points). The total score of the CERAD-K was calculated by adding the scores of these 6 subtests. The CDR was calculated as the sum of all 6 items (memory, orientation, judgment and problem solving, community affairs, home and hobbies, and personal care) in the CDR scale. Composite rating consisted of 5 levels: 0 (none), 0.5 (questionable), 1 (mild), 2 (moderate), and 3 (severe) [18].

#### 2.2.4. ^11^C-Pittsburgh Compound B (PiB)-PET Image Acquisition and Processing

All participants underwent simultaneous 3D PiB-PET and 3D T1-weighted magnetic resonance imaging (MRI) scans with a 3.0 T Biograph mMR (PET-MR) scanner Siemens Healthcare, Erlangen, Germany) according to the manufacturer’s approved guidelines. PiB-PET was acquired 40 min after an intervention injection of 555 MBq of PiB (range 450–610 MBq). T1-weighted images were acquired in the sagittal orientation using the following parameters: repetition time = 1670 ms, echo time = 1.89 ms, field of view = 250 mm, 256 × 256 matrix, and slice thickness = 1.0 mm.

All image processing and data analyses were performed using statistical parametric mapping (SPM) (SPM8 software; Wellcome Centre for Human Neuroimaging, University College London, London, UK) in MATLAB (MathWorks, Natick, MA, USA). Static PiB-PET images were co-registered to individual T1 images and normalized into standard space using transformation parameters of T1 images to a standard Montreal Neurological Institute (MNI) template. Using Individual Brain Atlases using Statistical Parametric Mapping (IBASPM, http://www.thomaskoenig.ch/Lester/ibaspm.htm) software, inverse transformation parameters were used to bring an Automated Anatomical Labeling (AAL) 116 atlas [19] in a standard space to an individual space for each subject (resampling voxel size = 1 mm × 0.98 mm × 0.98 mm). Non-gray matter portions of the atlas were individually masked using the cerebral gray matter segment image of each subject. Using individual AAL116 atlases, mean regional PiB uptake values from cerebral regions were extracted from T1-co-registered PiB-PET images. The cerebellar gray matter was used as the reference region for quantitative normalization of cerebral PiB uptake value due to its relatively low Aβ deposition [20]. To measure PiB uptake in cerebellar gray matter regions, a probabilistic cerebellar atlas (Institute of Cognitive Neuroscience, University College London, London, UK; Cognitive Neuroscience Laboratory, Royal Holloway of London Egham, Surrey, UK) was brought into individual space as described earlier. Of 28 anatomical structural regions in the cerebellar atlas, the cerebellar lobular regions (except for the vermis) were used to extract mean cerebellar uptake values.

The AAL algorithm and a regional combining method [21] were applied to set regions of interest (ROIs) to characterize PiB retention levels in frontal, lateral parietal, precuneus/posterior cingulate cortex (PC/PCC), and lateral temporal regions where prominent PiB retention was reported [22]. Standardized uptake value ratios (SUVRs) for ROIs were calculated by dividing the mean value of all voxels within each ROI by mean cerebellar uptake value in the same image. Additionally, a global cortical ROI consisting of 4 ROIs was defined and a global cortical SUVR was generated by dividing the mean value of all voxels of the global cortical ROI by the mean cerebellar uptake value in the same image (Appendix A). Global cerebral Aβ deposition was defined as mean PiB retention value of global cortical ROI.

### 2.3. Statistical Analyses

All statistical analyses were performed using SPSS 22.0 (IBM Corporation, Chicago, IL, USA). Basic characteristics of the study sample were stratified by MCI. Cognitive frailty groups were compared using *χ*^2^ test (categorical variables) or Student’s *t*-test (continuous variables). SUVRs between subjects with MCI and cognitive frailty were determined using independent sample *t*-test for normally distributed variables (SUVR of frontal cortex, temporal cortex, parietal cortex, PC/PCC, hippocampus, basal ganglia, and global SUVR). The proportion of subjects with positive findings in visual analysis was calculated for each group, and the percentage of cases in which the visual analysis result was consistent in ^11^C-PiB-PET images was calculated. Univariable and multivariable (adjusted for age and level of education) logistic regressions exploring the association between SUVRs for PiB-PET and physical frailty and/or physical function were performed. Statistical significance was set at *p* < 0.05.

## 3. Results

### 3.1. Baseline Characteristics

At baseline, the mean age of study participants was 75.1 ± 6.55 (SD) years. Of 48 subjects, 35 (73%) were female; mean years of education for all subjects was 9.4 ± 4.20 years. Table 1 presents the characteristics of MCI + Robust (43.8% of total) and cognitive frailty (56.3%) patients. Differences were significant in terms of slow gait velocity (*p* = 0.034), weakness (*p* = 0.000), exhaustion (*p* = 0.009), and low activity level (*p* = 0.000) between the two groups. Construction, mean (SD), and execution for CERAD-K also showed significant differences between the MCI + Robust and cognitive frailty groups (Table 1).

### 3.2. Quantitative and Visual Analysis Comparing SUVR between MCI and Cognitive Frailty Groups

Mean SUVRs according to brain regions in the MCI + Robust and cognitive frailty groups are summarized in Table 2. SUVRs for ^11^C-PiB-PET were not significantly different between the two groups (Table 2). Typical negative images in an MCI + Robust subject and positive images in a patient with cognitive frailty are shown in Appendix A. After adjusting the window level of ^11^C-PiB-PET with reference to the cerebellar cortex, each case failed to show significantly higher white matter uptake than gray matter uptake in the MCI + Robust and cognitive frailty groups. Overall, all participants showed identical results based on visual analysis of both PET images (Appendix B).

### 3.3. Association between SUVR and Measures of Physical Function by Brain Region

**Figure 1:**
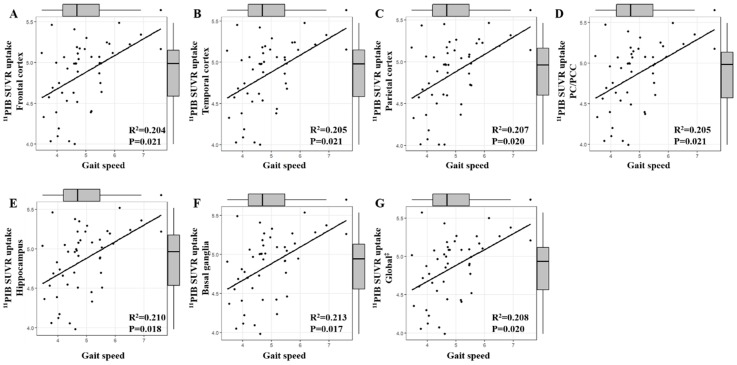
Association between mean Standardized uptake value ratio (SUVR) and gait speed by brain region. Arithmetic mean of frontal cortex, lateral temporal cortex, parietal cortex, precuneus/posterior cingulate cortex (PC/PCC), and basal ganglia SUVR. Adjusted for age and level of education.

Global amyloid burden (SUVR) was significantly linked to gait speed (*p* = 0.020), basal ganglia (*p* = 0.017), PC/PCC (*p* = 0.021), parietal cortex (*p* = 0.020), temporal cortex (*p* = 0.021), frontal cortex (*p* = 0.021), and hippocampus (*p* = 0.018) (Figure 1). In addition, basal ganglia and PC/PCC were significantly linked to SPPB (*p* = 0.047 and *p* = 0.043, respectively) and TUG (*p* = 0.033 and *p* = 0.026, respectively).

### 3.4. Association between SUVR and Physical Frailty by Brain Region

SUVRs of all brain regions were significantly linked to weakness as follows: global amyloid burden (*p* = 0.009), frontal cortex (*p* = 0.010), temporal cortex (*p* = 0.008), parietal cortex (*p* = 0.023), PC/PCC (*p* = 0.009), hippocampus (*p* = 0.008), and basal ganglia (*p* = 0.009) (Table 3).

## 4. Discussion

In this study, we examined the association between amyloid-β accumulation in the brain assessed with a brain imaging biomarker and physical frailty (weight loss, weakness, exhaustion, slowness, and low physical activity) in older adults with MCI and cognitive frailty. To the best of our knowledge, this is one of the first studies describing the relationship between brain amyloid load and physical frailty in humans. The major finding of this study is that there was a significant association between gait speed and brain amyloid-β accumulation (measured with ^11^C-PiB-PET) in the temporal cortex, parietal cortex, PC/PCC, and basal ganglia. In addition, SUVRs of all brain regions were significantly linked to weakness.

In visual analysis, the cortical uptake of ^11^C-PiB was not significantly different between the MCI + Robust group and the cognitive frailty group. Quantitative analysis showed that 11C-PiB global SUVR did not significantly vary between MCI + Robust patients and cognitive frailty patients, in agreement with previous studies showing cortical SUVR measured with ^11^C-PiB-PET in patients with MCI and AD [23]. However, few studies have shown that Aβ deposition occurs in normal elderly subjects who might subsequently develop signs of MCI and ultimately develop AD. Postmortem analysis has demonstrated a characteristic abundance of Aβ plaques in specific brain areas of AD patients. Recent PET studies using ^11^C-PiB in elderly normal subjects support the existence of a preclinical AD stage in which Aβ plaques are found in discrete brain regions based on significant radio ligand retention in about 10% of the control subjects, approaching levels seen in AD subjects [24].

However, the strongest association has been observed between ^11^C-PiB-PET global SUVRs (temporal cortex, parietal cortex, PC/PCC, basal ganglia, frontal cortex, and hippocampus) and gait speed, SPPB (PC/PCC, basal ganglia), and TUG (PC/PCC, basal ganglia), with analysis of each individual’s data instead of a group comparison. Similarly, previous studies have shown that ventricular enlargement in the temporal horn is associated with worse gait parameters, including variation in stride time and gait speed [9]. In addition, a recent MAPT study reported that the usual pace of walking is associated with amyloid deposition assessed by PET scans [8]. These authors found a significant association between amyloid in posterior and anterior putamen, occipital cortex, precuneus, and anterior cingulate and slow gait speed. Gait speed is a marker of frailty phenotypes attributed to an age-related reduction in physiologic reserves [12]. Studies have consistently shown that gait speed can predict major health-related events, including future disability, hospitalization, and death [25]. In addition, both cognition and APOE ε4 genotype may influence the association between Aβ and gait speed in dementia-free older adults [10]. However, as a limitation, APOE carrier status was not initially considered and thus was not determined in our study. Thus, slow gait speed is potentially influenced by unknown mechanisms that may increase the susceptibility of the brain with MCI to AD pathology.

A major contribution of this study involves the assessment of global SUVR in participants with cognitive frailty. In the present study, the association between brain amyloid-β and weakness was reflected by SUVRs of all brain regions. Recent studies have shown that muscle weakness based on handgrip strength evaluation can predict exhaustion, functional decline, morbidity, and mortality. In fact, frailty is probably sixfold higher among persons with reduced grip strength [26]. Muscle weakness has also been indicated as one of the initial manifestations of frailty [26]. Nevertheless, in the absence of studies investigating the association between brain amyloid level and chair rise, it is impossible to compare our results with the published literature. It is known that patients with AD exhibit increased levels of amyloid deposition in brain and muscle, and that intracellular amyloid-β in muscle fibers is associated with reduced muscle strength and potential sarcopenia [9]. Our previous study using data from a MAPT population found negative associations between gait speed and regional brain amyloid [8]. We hypothesized that the magnitude of amyloid-β levels in the brain would be associated with frailty conditions. However, based on our results, only grip strength in frailty index was significantly associated with amyloid-β levels in different brain regions.

The present study is limited by a relatively small sample size and including subjects at a single institution. A multi-institutional study involving a larger number of subjects is required to corroborate the results of this study.

## 5. Conclusions

Overall, this study shows that amyloid levels in brain cortices or regions are not associated with each other in patients with MCI and cognitive frailty. Nevertheless, the present study found a correlation between brain amyloid-β levels and weakness based on SUVRs of different brain regions. Furthermore, an association exists between global SUVR (frontal cortex, temporal cortex, parietal cortex, PC/PCC, hippocampus, and basal ganglia) and gait. However, additional studies are needed to confirm or refute our findings. Further research is needed to elucidate neural mechanisms underlying this association, ideally involving exercise interventions and study designs that facilitate analysis of causal relationships.

## Figures and Tables

**Table 1:** Baseline characteristics of study population.

Variable	Full Sample, *n* = 48	Physical Frailty Status		*p* Value
		MCI + Robust, *n* = 21 (43.8%)	Cognitive Frailty, *n* = 27 (56.3%)	
Demographics				
Age, mean (SD)	75.1 (6.55)	74.6 (5.65)	75.5 (7.28)	0.644
Female, n (%)	35 (73%)	14 (67%)	21 (78%)	0.285
Education, y, mean (SD)	9.4 (4.20)	9.1 (4.12)	9.7 (4.33)	0.665
Frailty criteria, n (%)				
Slow gait velocity	5 (10.2%)	0	5 (19%)	0.034
Shrinking	4 (8.2%)	0	4 (15%)	0.061
Weakness	13 (26.5%)	0	13 (48%)	<0.001
Exhaustion	7 (14.3%)	0	7 (26%)	0.009
Low activity level	11 (22.4%)	0	11 (41%)	<0.001
Cognitive functioning				
MMSE (score), mean (SD)	24.3 (2.31)	24.7 (2.46)	24.0 (2.19)	0.285
Cognitive impairment (MMSE <23), n (%)	18 (36.7%)	6 (27.3%)	13 (48.1%)	0.142
CERAD-K				
Memory, mean (SD)	25.0 (5.74)	24.5 (5.97)	25.4 (5.63)	0.622
Construction, mean (SD)	9.7 (1.44)	10.2 (1.33)	9.3 (1.44)	0.039
Execution, mean (SD)	13.3 (4.58)	14.9 (4.59)	12.1 (4.25)	0.035
Naming, mean (SD)	10.0 (2.43)	10.1 (2.37)	9.9 (2.53)	0.816
Total score, mean (SD)	58.0 (10.06)	59.7 (10.63)	56.6 (9.56)	0.296

MCI: Mild cognitive impairment, SD: standard deviation, MMSE: mini mental state examination, CREAD-K: A Korean version of the consortium to establish a Registry for Alzheimer’s Disease Assessment.

**Table 2:** Comparison of standard uptake value ratios (SUVRs) for 11C-Pittsburgh compound B (^11^C-PiB) in MCI and cognitive frailty subjects.

	MCI + Robust	Cognitive Frailty	*p* Value
Frontal cortex	1.28 ± 0.41	1.47 ± 0.54	0.371
Temporal cortex	1.24 ± 0.35	1.40 ± 0.50	0.433
Parietal cortex	1.27 ± 0.43	1.44 ± 0.54	0.438
PC/PCC	1.43 ± 0.46	1.63 ± 0.60	0.424
Hippocampus	1.22 ± 0.21	1.27 ± 0.16	0.330
Basal ganglia	1.37 ± 0.37	1.43 ± 0.35	0.560
Global ^‡^	1.32 ± 0.39	1.41 ± 0.40	0.429

^‡^ Arithmetic mean of frontal cortex, lateral temporal cortex, parietal cortex, precuneus/posterior cingulate cortex (PC/PCC), and basal ganglia SUVR.

**Table 3:** Association between mean SUVR and frailty index criteria by brain region.

	Weight Loss		Exhaustion		Weakness		Slowness		Low Activity	
	β	*p*	β	*p*	β	*p*	β	*p*	β	*p*
Frontal cortex	−0.149	0.312	0.072	0.627	0.367	0.010	−0.033	0.821	−0.023	0.877
Temporal cortex	−0.138	0.350	−0.010	0.345	0.377	0.008	−0.003	0.986	−0.020	0.895
Parietal cortex	−0.179	0.223	0.076	0.609	0.328	0.023	0.000	0.997	−0.035	0.811
PC/PCC	−0.144	0.327	0.049	0.742	0.372	0.009	0.030	0.837	−0.017	0.911
Hippocampus	0.018	0.905	−0.086	0.563	0.377	0.008	0.030	0.841	−0.010	0.946
Basal ganglia	−0.104	0.482	−0.047	0.753	0.374	0.009	0.011	0.943	−0.030	0.842
Global ^‡^	−0.148	0.316	0.033	0.823	0.371	0.009	0.002	0.991	−0.025	0.864

β, completely standardized regression coefficient; ^‡^, arithmetic mean of frontal cortex, lateral temporal cortex, parietal cortex, precuneus/posterior cingulate cortex (PC/PCC), and basal ganglia SUVR.

**Figure A1:**
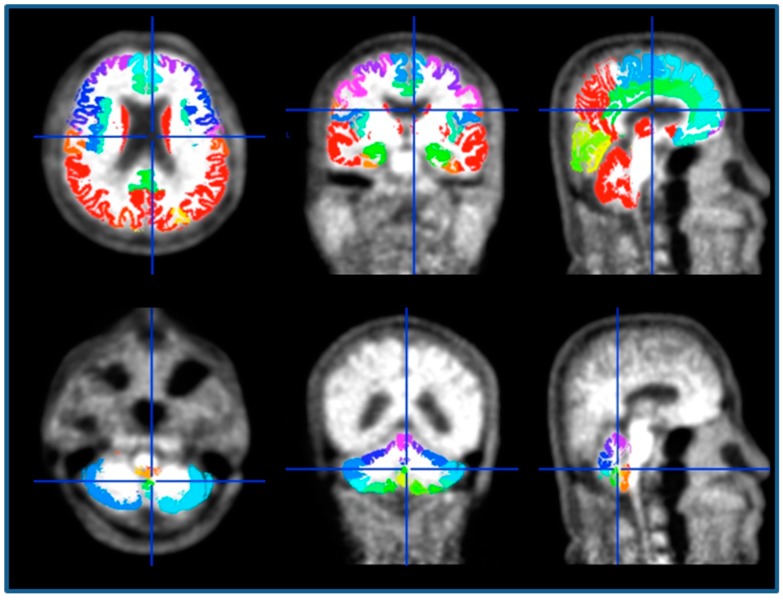
Representative images of regions of interest based on Automatic Anatomic Labeling (AAL) for individual ^11^C-PiB-PET data. Using the published list of automatically labeled regions [19], the frontal regions of interest (ROIs) included bilateral frontal, anterior cingulate, middle cingulate, and insular cortex regions (region 3–34); the posterior cingulate-precuneus ROIs included bilateral regions (region 35;36) and (region 67;68); the lateral temporal ROIs included bilateral regions (region 79–90); the lateral parietal ROIs included bilateral regions (region 59–65); the basal ganglia ROIs included bilateral caudate, putamen, and globus pallidus regions (region 71–76); the medial temporal ROIs included bilateral hippocampus, parahippocampal gyrus, and amygdala regions (region 37–42); and the cerebellar reference ROIs included bilateral cerebellar crus I regions (region 91;92) [21].

**Figure A2:**
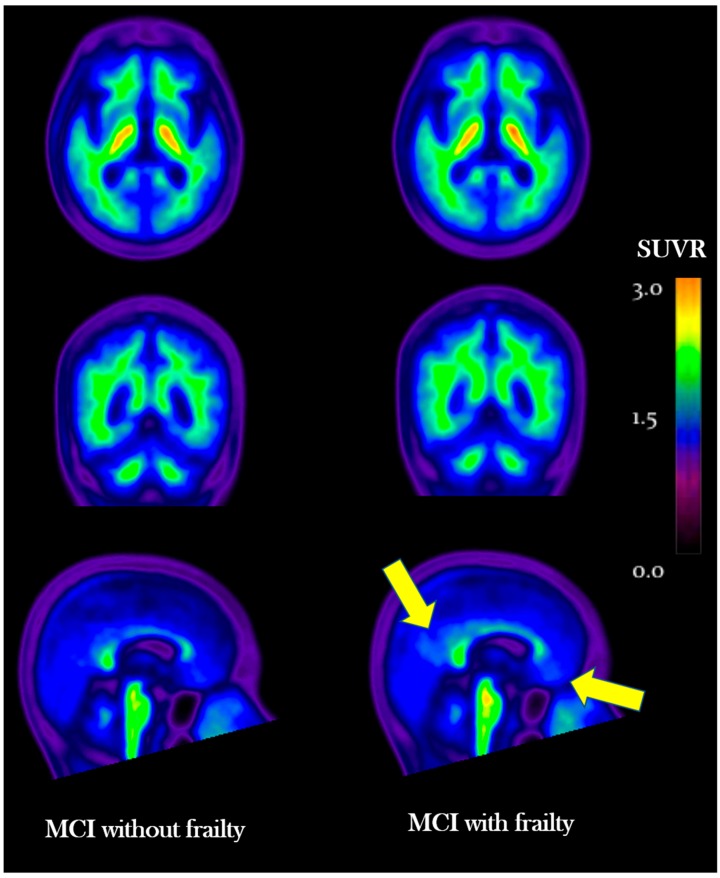
Representative images of ^11^C-PiB-PET for cerebral amyloid burden of the same MCI and the cognitive frailty patients. Images scaled to the same SUVR are shown. Left row: negative amyloid scan; right row: positive amyloid scan. SUVR = standard uptake value ratio.
